# Bridging Critically Ill Patients With Cirrhosis to Transplant With Renal Replacement Therapy: A Multicenter Cohort Study

**DOI:** 10.1111/liv.70593

**Published:** 2026-03-18

**Authors:** Filipe S. Cardoso, Minjee Kim, Beverley Kok, Richard Wunderink, Juan G. Abraldes, Constantine J. Karvellas

**Affiliations:** ^1^ Transplant Unit Curry Cabral Hospital, Nova Medical School Lisbon Portugal; ^2^ Division of Neurocritical Care, Department of Neurology Northwestern University Feinberg School of Medicine Chicago Illinois USA; ^3^ Liver Unit Royal Free Hospital London UK; ^4^ Department of Medicine Northwestern University Feinberg School of Medicine Chicago Illinois USA; ^5^ Liver Unit University of Alberta Hospital Edmonton Canada; ^6^ Department of Critical Care University of Alberta Hospital Edmonton Canada

**Keywords:** death, dialysis, liver failure, renal insufficiency, transplantation

## Abstract

**Background & Aims:**

The efficacy of renal replacement therapy (RRT) in critically ill patients with cirrhosis remains dubious. We aimed to assess the impact of RRT on these patients' outcomes.

**Methods:**

Multicenter retrospective cohort study including adult patients with cirrhosis admitted to intensive care units at University of Alberta Hospital (Edmonton, Canada) and Northwestern Memorial Hospital (Chicago, US) from January 2010 to December 2017. Primary exposure was receipt of RRT on ICU days 1 to 3. Fine and Gray multivariable regression with competing endpoints, in‐hospital liver transplant (LT) and mortality, was performed.

**Results:**

Among 898 patients, median (IQR) age was 57 (49–64) years and 539 (60.0%) were males. RRT on days 1 to 3 was used in 249 (27.7%) patients. Patients on RRT on days 1 to 3 had higher CLIF‐C‐ACLF scores on days 1 (61 vs. 55, *p* < 0.001) and 3 (59 vs. 50; *p* < 0.001) than others. During the hospital stay, 97 (10.8%) patients were transplanted and 296 (33.0%) died. Following adjustment for aetiology, number of extra‐renal organ failures, and year of inclusion, using mortality as competing event, RRT on days 1 to 3 was associated with higher hazard of LT (HR (95% CI) = 1.54 (1.02–2.32); *p* = 0.039). Conversely, using LT as competing event, RRT on days 1 to 3 was not associated with mortality (HR (95% CI) = 1.15 (0.90–1.47); *p* = 0.25).

**Conclusions:**

Among critically ill patients with cirrhosis, early RRT, while offered more often to the sickest patients, was associated with a higher likelihood of receiving LT, but not with mortality.

## Key Points

- On intensive care unit (ICU) days 1 to 3, about a quarter of patients with cirrhosis required renal replacement therapy (RRT).
- Patients on RRT in ICU days 1 to 3 had higher overall severity of disease than others.
- Following adjustment for aetiology, severity of disease, and year of inclusion, being on RRT on days 1 to 3 was independently associated with a higher hazard of being transplanted during the hospital stay.
- The receipt of RRT on days 1 to 3 did not impact in‐hospital mortality.

**Abbreviations**
ACLF: acute‐on‐chronic liver failure
AKI: acute kidney injury
ALD: alcohol‐associated liver disease
BMI: body mass index
CI: confidence interval
CLIF‐C‐ACLF: Chronic Liver Failure Acute‐on‐Chronic Liver Failure score
HCV: hepatitis C virus
HE: hepatic encephalopathy
HR: hazard ratio
ICU: intensive care unit
INR: international normalised ratio
IQR: interquartile range
LOS: length‐of‐stay
LT: liver transplant
MELD: Model for End‐stage Liver Disease
PF: arterial oxygen pressure/inspired oxygen fraction ratio
RRT: renal replacement therapy
sCr: serum creatinine
TFS: transplant‐free survival

## 1 Introduction

Among patients with cirrhosis, the management of acute kidney injury (AKI) remains a challenge. Overtime, patients with cirrhosis have been gaining better access to critical care [1]. More often, clinicians grant these patients a trial of at least 48 to 72 h of organ support, especially while potential liver transplant (LT) candidacy is considered. However, their in‐hospital overall survival may still be lower than 50% [2].

In the intensive care unit (ICU), more than 40% of patients with cirrhosis may develop AKI [3]. Its more frequent causes have been reported to be sepsis, hypovolemia, hepato‐renal syndrome (HRS), and parenchymal disease [4, 5]. Often, in this context, more than one of these disease mechanisms may contribute concomitantly to AKI, making its differential diagnosis and prognosis harder to establish. However, despite the underlying mechanism, the initial treatment of critically ill patients with cirrhosis and AKI frequently relies on resuscitation using intravenous fluids and/or vasopressors [6].

Among patients with cirrhosis in the ICU, more than 15% may require renal replacement therapy (RRT) [6, 7]. In this context, frequent indications for RRT include AKI with life‐threatening metabolic derangements, hypervolemia, hyponatremia, or hyperammonemia. Among these patients, the need for RRT has been associated with lower all‐cause one‐year survival [8, 9]. However, as RRT in this context may simply reflect the greater severity of kidney failure or overall disease, doubts remain about the potential impact of RRT on these patients' outcomes [10, 11]. Moreover, clinicians often face the difficult balance between assessing the likelihood of 2 different outcomes—improving these patients' condition until LT or dying without transplant.

Accordingly, we hypothesised that, among critically ill patients with cirrhosis, RRT may contribute to modify their outcomes, especially access to LT. Therefore, the objectives of this study were the following: (1) describe the evolving prevalence of RRT use among patients with cirrhosis in the ICU; (2) assess the potential association between RRT and in‐hospital LT and survival.

## 2 Methods

### 2.1 Design, Setting, Participants, and Ethics

This was a multi‐center retrospective observational cohort study. Consecutive adult (≥ 18 years) patients with cirrhosis admitted to one ICU at the University of Alberta Hospital (Edmonton, Canada) or 5 ICUs (for at least 48 h) at the Northwestern Memorial Hospital (Chicago, Illinois, US), between January 2010 and December 2017, were included. Patients were excluded if they had a previous liver transplant (LT) or were admitted to the ICU following elective LT. Among patients who were admitted to the ICU more than once during the inclusion period, only the first ICU admission meeting the above inclusion and exclusion criteria was considered in this analysis. Data retrieved for this study has been at least partially used in 2 previous studies [12, 13].

As this was a noninterventional and anonymised study, the institutional review boards of both centers waived the need for individual informed consent (Pro00035429, 04/02/2013, University of Alberta Health Research Ethics Board; STU00204868, 08/11/2017, Northwestern University Institutional Review Board). All study procedures followed the principles of the Declaration of Helsinki [14]. The reporting of this study followed the Strengthening the Reporting of Observational Studies in Epidemiology (STROBE) guideline [15].

### 2.2 Definitions, Data Retrieval, Exposures, and Endpoints

Cirrhosis was defined as bridging fibrosis on liver biopsy or a composite of clinical signs and findings provided by laboratory tests, endoscopy, and radiologic imaging [12, 13].

Organ failures, Acute‐on‐chronic liver failure (ACLF), Chronic Liver Failure Sequential Organ Failure Assessment (CLIF‐SOFA) score, and CLIF‐C‐ACLF score were defined based on the European Foundation for the Study of Chronic Liver Failure (CLIF) Consortium criteria [16]. Model for end‐stage liver disease (MELD) score was defined as the original equation considering only serum INR, bilirubin, and creatinine [17].

As baseline serum creatinine levels were not available, AKI had to be defined based on a serum creatinine of ≥ 1.5 mg/dL on days 1 and 3 post ICU admission, a cutoff frequently used in patients with cirrhosis [18]. In this context, to avoid overestimating the AKI diagnosis, patients on dialysis prior to ICU admission did not qualify for AKI criteria despite their serum creatinine levels or RRT use on days 1 and 3 post ICU admission [19]. Urine output data were not reliably available to inform AKI diagnosis.

The following baseline characteristics of patients were retrieved from the electronic health records: age, sex, and body mass index; cirrhosis aetiology; hepatocellular carcinoma; dialysis prior to hospital admission; precipitant event leading to ICU admission (infection as clinical or culture‐based diagnosis and bleeding as clinical or haemoglobin < 70 g/L diagnosis); organ failures and support on days 1 and 3 post ICU admission (as per CLIF‐SOFA score), general blood biochemistry on days 1 and 3 post ICU admission; severity of disease scores on ICU days 1 and 3, namely CLIF‐C‐ACLF and MELD scores [16, 17]; and year of enrollment (stratified as 2010–2013 vs. 2014–2017 because the landmark Canonic study on ACLF was published in 2013). Additionally, data on the following outcomes was also retrieved: index ICU and hospital length‐of‐stay (LOS); and index in‐hospital LT, all‐cause mortality, and transplant‐free survival (TFS) rates.

The primary exposure was RRT prescribed at local clinicians' discretion during the first 3 days following ICU admission. As clinicians may have different approaches to RRT initiation, reflecting variability within the literature on this field of expertise, we chose to include any RRT deployment up to 3 days following ICU admission to better capture its use in this context [20]. We captured both continuous RRT (delivered for 24 h per day) and intermittent RRT (delivered most often up to 4–6 h per day) use in the ICUs. Data on further RRT specifics, such as the venous access type, delivery modalities, dialysis or filtration doses, replacement fluids, downtime, or termination rules were not available.

The endpoints assessed were index in‐hospital LT, all‐cause mortality, and TFS. We chose these endpoints because we aimed to study primarily if RRT could modify LT access for these patients, irrespective of the likelihood of death; moreover, for this purpose, we would need to account for LT and mortality as potential competing events (with TFS as the censored event). Transplant eligibility criteria were center specific, although based on international recommendations [21, 22]. After ICU admission, patients were followed up until death or hospital discharge, whichever came first.

### 2.3 Statistical Analysis

Continuous and categorical variables were described as median [interquartile range (IQR)] and frequency (%), respectively. Overall missing data across all values was 7.5% and no imputation was performed. Univariate comparisons were performed using the Mann–Whitney and chi‐square tests where appropriate.

Multivariable analysis was performed with cox proportional hazards regression. Time‐to‐event analysis was preferred because, in critically ill patients with cirrhosis, time to LT has been shown to influence outcomes [21, 22]. Moreover, competing risks between LT and mortality could add to this analysis. To minimise the occurrence of immortal time bias, we excluded the 16 patients that died during the first 3 days of ICU stay for the multivariable analyses.

The development of the final models initially included variables deemed clinically significant and/or with a *p* value < 0.10 on the univariable comparisons. Collinearity or interaction was avoided where appropriate. The models' further development used a backward stepwise elimination process, with the final models yielding the best fit. Finally, a time‐to‐event analysis, with competing risk, was performed using the univariable cumulative incidence function and the Fine and Gray multivariable approach [23, 24].

As aetiology seemed to be associated with outcomes, and alcohol‐associated liver disease was the single most frequent one, we decided to code this variable as alcohol vs. other. To avoid collinearity or interaction, we decided to use the overall number of organ failures as a surrogate for acuity [3, 6]. This metric was considered on ICU day 1 as this timing has been repeatedly linked with greater overall severity of disease during the hospital stay [6, 16]. Based on the CLIF‐SOFA score, the overall number of organ failures included parameters such as HE grade, PF ratio, vasopressors, INR, bilirubin, and creatinine or RRT [16]. To single out RRT on ICU days 1–3, the exposure of interest, we used the added number of organ failures but without the kidney component. As the study enrollment comprised 8 years, we also included year of enrollment in the models.

The statistical significance was set as *p* < 0.05 (2‐tailed). The statistical analyses were performed using IBM SPSS Statistics, version 29 (IBM Corp, North Castle, NY, US) and R, version 4.2.2 (R Foundation for Statistical Computing, Vienna, Austria), with the cmprsk package.

## 3 Results

### 3.1 Baseline Characteristics

Among 898 patients with cirrhosis admitted to the ICU, median (IQR) age was 57 (49–64) years and 539 (60.0%) were males. Alcohol‐associated liver disease (ALD) was the leading aetiology of cirrhosis, as it was present alone in 367 (40.9%) patients and in combination with hepatitis C in 85 (9.5%) additional patients (Table 1).

**TABLE 1:** Baseline characteristics and outcomes of critically ill patients with cirrhosis stratified by renal replacement therapy status.

Characteristic	Total (*n* = 898)	RRT on days 1–3 (*n* = 249)	No RRT (*n* = 649)	*p*
Age (years) (*n* = 897)	57 (49–64)	58 (51–64)	56 (48–63)	0.11
Sex (male)	539 (60.0%)	151 (60.6%)	388 (59.8%)	0.81
BMI (kg/m^2^) (*n* = 590)	28 (24–33)	28 (25–33)	28 (24–33)	0.28
Cirrhosis aetiology				0.10
Alcohol	367 (40.9%)	91 (36.5%)	276 (42.5%)	
HCV	94 (10.5%)	23 (9.2%)	71 (10.9%)	
Alcohol + HCV	85 (9.5%)	21 (8.4%)	64 (9.9%)	
Others	352 (39.2%)	114 (45.8%)	238 (36.7%)	
Hepatocellular carcinoma	72 (8.0%)	18 (7.2%)	54 (8.3%)	0.59
Prior dialysis (*n* = 895)	41 (4.6%)	21 (8.4%)	20 (3.1%)	< 0.001
Precipitant event				0.17
Infection	357 (39.8%)	100 (40.2%)	257 (39.6%)	
Bleeding	74 (8.2%)	18 (7.2%)	56 (8.6%)	
Infection + bleeding	76 (8.5%)	29 (11.6%)	47 (7.2%)	
Other	391 (43.5%)	102 (41.0%)	289 (44.5%)	
General organ function and support on day 1				
Grade 3/4 HE	321 (35.7%)	85 (34.1%)	236 (36.4%)	0.53
Ventilation	489 (54.5%)	131 (52.6%)	358 (55.2)	0.49
PF ratio (mmHg) (*n* = 564)	200 (137–280)	188 (122–286)	203 (144–280)	0.39
Vasopressors (*n* = 853)	370 (43.4%)	134 (55.8%)	236 (38.5%)	< 0.001
MAP (*n* = 597)	58 (50–66)	57 (48–66)	58 (51–66)	0.25
INR (*n* = 880)	1.9 (1.5–2.5)	2.1 (1.6–3.0)	1.9 (1.5–2.4)	< 0.001
Bilirubin (mg/dL) (*n* = 878)	4.9 (2.1–11.8)	6.8 (2.5–17.3)	4.3 (1.9–10.1)	< 0.001
Lactate (mmol/L) (*n* = 694)	2.6 (1.7–4.9)	3.1 (1.9–6.7)	2.4 (1.6–4.6)	< 0.001
Sodium (mmol/L) (*n* = 753)	135 (132–139)	134 (131–138)	136 (132–140)	< 0.001
pH (*n* = 673)	7.34 (7.26–7.40)	7.29 (7.20–7.37)	7.36 (7.28–7.41)	< 0.001
Kidney function and support on days 1–3				
sCr on day 1 (mg/dL) (*n* = 891)	1.69 (1.06–3.04)	3.15 (1.95–4.51)	1.43 (0.96–2.29)	< 0.001
AKI on day 1 (*n* = 891)	515 (57.8%)	228 (91.6%)	287 (44.7%)	< 0.001
sCr on day 3 (mg/dL) (*n* = 727)	1.38 (0.88–2.18)	1.86 (1.24–3.05)	1.22 (0.78–1.84)	< 0.001
AKI on day 3 (*n* = 727)	410 (55.0%)	225 (95.3%)	185 (36.3%)	< 0.001
Severity of disease on days 1–3				
Organ failures on day 1 (0 to 6)	2 (1–3)	3 (2–4)	1 (1–3)	< 0.001
CLIF‐C‐ACLF on day 1 (*n* = 844)	56 (49–64)	61 (52–68)	55 (47–62)	< 0.001
MELD on day 1 (*n* = 870)	27 (19–35)	36 (30–42)	23 (16–31)	< 0.001
Organ failures on day 3 (0 to 6) (*n* = 882)	1 (0–3)	3 (1–4)	1 (0–2)	< 0.001
CLIF‐C‐ACLF on day 3 (*n* = 698)	52 (44–60)	59 (49–68)	50 (43–57)	< 0.001
MELD on day 3 (*n* = 542)	27 (19–36)	37 (31–43)	22 (15–29)	< 0.001
In‐hospital outcomes				
LT	97 (10.8%)	39 (15.7%)	58 (8.9%)	0.004
Time to LT (days)	12 (6–20)	10 (6–19)	13 (6–20)	0.48
All‐cause mortality	296 (33.0%)	101 (40.6%)	195 (30.0%)	0.003
Time to death (days)	15 (8–28)	15 (6–29)	15 (9–28)	0.23
Transplant‐free survival	510 (56.8%)	110 (44.2%)	400 (61.6%)	< 0.001
ICU LOS (days)	6 (3–12)	7 (4–14)	5 (3–11)	< 0.001
Hospital LOS (days) (*n* = 896)	15 (9–27)	15 (8–28)	15 (9–27)	0.50
Year of enrollment				0.33
2010–2013	413 (46.0%)	108 (43.4%)	305 (47.0%)	
2014–2017	485 (54.0%)	141 (56.6%)	344 (53.0%)	

Abbreviations: AKI, acute kidney injury; BMI, body mass index; CLIF‐C‐ACLF, Chronic Liver Failure Acute‐on‐Chronic Liver Failure score; HCV, hepatitis C virus; HE, hepatic encephalopathy (West Haven criteria); ICU, intensive care unit; INR, international normalised ratio; LOS, length‐of‐stay; LT, liver transplant; MELD, Model for End‐stage Liver Disease; PF, arterial oxygen pressure/inspired oxygen fraction ratio; RRT, renal replacement therapy; sCr, serum creatinine; TFS, transplant‐free survival.

The most frequent precipitant event leading to decompensation of cirrhosis and ICU admission was infection, which was diagnosed alone in 357 (39.8%) patients and, in conjunction with bleeding, in 76 (8.5%) added patients (Table 1).

From ICU days 1 to 3, the median (IQR) number of organ failures decreased from 2 (1–3) to 1 (0–3), respectively. The ACLF grading on ICU days 1 and 3 is detailed in Table S1.

From ICU days 1 to 3, the median (IQR) CLIF‐C‐ACLF scores decreased from 56 (49–64) to 52 (44–60). However, the median (IQR) MELD scores were similar—27 (19–35) and 27 (19–36), respectively (Table 1).

### 3.2 General Outcomes

Among all patients included, 97 (10.8%) were transplanted within the index hospital stay, with a median (IQR) time to transplant of 12 (6–20) days (maximum of 44 days). During the index hospital stay, 510 (56.8%) survived without transplant. Median (IQR) time to death was 15 (8–28) days (maximum of 151 days). From ICU days 1 to 3, 16 (1.8%) patients died (Table 1).

### 3.3 Acute Kidney Injury and Renal Replacement Therapy

AKI was diagnosed on ICU days 1 and 3 in 515 (57.8%) and 410 (55.0%) patients respectively. RRT was offered to 249 (27.7%) patients on ICU days 1–3, with 197 (79.1%) using continuous modalities. Patients who received RRT on ICU days 1–3 had more often dialysis before the index ICU admission than others (8.4% vs. 3.1%; *p* < 0.001) (Table 1).

On ICU day 1, patients on RRT on ICU days 1–3 had more vasopressors (55.8% vs. 38.5%; *p* < 0.001), worse liver function (median INR and bilirubin of 2.1 vs. 1.9 and 6.8 vs. 4.3 mg/dL, respectively; *p* < 0.001 for both), more severe hyperlactatemia (median lactate of 3.1 vs. 2.4 mmol/L; *p* < 0.001), more hyponatremia (median sodium of 134 vs. 136 mmol/L; *p* < 0.001), and worse acidosis (median pH of 7.29 vs. 7.36; *p* < 0.001) than others. Moreover, patients with RRT on ICU days 1–3 had more AKI on ICU days 1 (91.6% vs. 44.7%) and 3 (95.3% vs. 36.3%; *p* < 0.001 for both) than others (Table 1).

Patients who received RRT on ICU days 1–3 showed a higher overall severity of disease than others, detailed as follows: higher median number of organ failures on ICU days 1 (3 vs. 1) and 3 (3 vs. 1; *p* < 0.001 for both); higher median CLIF‐C‐ACLF scores on ICU days 1 (61 vs. 55) and 3 (59 vs. 50; *p* < 0.001 for both); and higher median MELD scores on ICU days 1 (36 vs. 23) and 3 (37 vs. 22; *p* < 0.001 for both) (Table 1).

Patients on RRT on ICU days 1–3 had higher prevalence of in‐hospital LT (15.7% vs. 8.9%; *p* = 0.004) and all‐cause mortality (40.6% vs. 30.0%; *p* = 0.003) than others. Thus, such patients on RRT had lower prevalence of in‐hospital TFS than others (44.2% vs. 61.6%; *p* < 0.001) (Table 1).

### 3.4 Univariable Analysis: Associations With In‐Hospital Liver Transplant

Patients who underwent LT during the index hospital stay had a lower prevalence of ALD (alone or with hepatitis C) than others (36.1% vs. 52.1%; *p* = 0.003). Moreover, transplanted patients had less respiratory failure on ICU day 1 (median arterial oxygen pressure/inspired oxygen fraction (PF) ratio of 220 vs. 198 mmHg; *p* = 0.036) and more need for RRT on ICU days 1–3 (40.2% vs. 26.2%; *p* = 0.004) than others (Table 2).

**TABLE 2:** Baseline characteristics and outcomes of critically ill patients with cirrhosis stratified by liver transplant status.

Characteristic	Total (*n* = 898)	In‐hospital LT (*n* = 97)	No LT (*n* = 891)	*p*
Age (years) (*n* = 897)	57 (49–64)	57 (49–63)	57 (49–64)	0.56
Sex (male)	539 (60.0%)	56 (57.7%)	483 (60.3%)	0.63
BMI (kg/m^2^) (*n* = 590)	28 (24–33)	29 (25–33)	28 (24–33)	0.19
Cirrhosis aetiology				0.011
Alcohol	367 (40.9%)	25 (25.8%)	342 (42.7%)	
HCV	94 (10.5%)	15 (15.5%)	79 (9.9%)	
Alcohol + HCV	85 (9.5%)	10 (10.3%)	75 (9.4%)	
Others	352 (39.2%)	47 (48.5%)	305 (38.1%)	
Hepatocellular carcinoma	72 (8.0%)	12 (12.4%)	60 (7.5%)	0.10
Prior dialysis (*n* = 895)	41 (4.6%)	3 (3.1%)	38 (4.8%)	0.46
Precipitant event				0.22
Infection	357 (39.8%)	34 (35.1%)	323 (40.3%)	
Bleeding	74 (8.2%)	10 (10.3%)	64 (8.0%)	
Infection + bleeding	76 (8.5%)	13 (13.4%)	63 (7.9%)	
Other	391 (43.5%)	40 (41.2%)	351 (43.8%)	
General organ function and support on day 1				
Grade 3/4 HE	321 (35.7%)	35 (36.1%)	286 (35.7%)	0.94
Ventilation	489 (54.5%)	50 (51.5%)	439 (54.8%)	0.54
PF ratio (mmHg) (*n* = 564)	200 (137–280)	220 (164–325)	198 (133–280)	0.036
Vasopressors (*n* = 853)	370 (43.4%)	38 (39.2%)	332 (43.9%)	0.38
MAP (*n* = 597)	58 (50–66)	57 (49–66)	58 (50–66)	0.82
INR (*n* = 880)	1.9 (1.5–2.5)	2.4 (2.1–3.3)	1.9 (1.5–2.4)	< 0.001
Bilirubin (mg/dL) (*n* = 878)	4.9 (2.1–11.8)	15.0 (7.2–27.3)	4.2 (1.9–9.6)	< 0.001
Lactate (mmol/L) (*n* = 694)	2.6 (1.7–4.9)	3.7 (2.0–5.6)	2.5 (1.7–4.8)	0.017
Sodium (mmol/L) (*n* = 753)	135 (132–139)	134 (130–138)	135 (132–139)	0.006
pH (*n* = 673)	7.34 (7.26–7.40)	7.37 (7.27–7.41)	7.34 (7.26–7.40)	0.34
Kidney function and support on days 1–3				
sCr on day 1 (mg/dL) (*n* = 891)	1.69 (1.06–3.04)	1.98 (1.16–3.43)	1.67 (1.05–3.00)	0.17
AKI on day 1 (*n* = 891)	515 (57.8%)	66 (68.0%)	449 (56.5%)	0.031
sCr on day 3 (mg/dL) (*n* = 727)	1.38 (0.88–2.18)	1.58 (0.95–2.62)	1.36 (0.86–2.14)	0.13
AKI on day 3 (*n* = 727)	410 (55.0%)	63 (67.0%)	347 (53.2%)	0.012
RRT on days 1–3	249 (27.7%)	39 (40.2%)	210 (26.2%)	0.004
Severity of disease on days 1–3				
Organ failures on day 1 (0 to 6)	2 (1–3)	3 (2–4)	2 (1–3)	< 0.001
CLIF‐C‐ACLF on day 1 (*n* = 844)	56 (49–64)	58 (49–64)	56 (50–64)	0.32
MELD on day 1 (*n* = 870)	27 (19–35)	34 (28–39)	25 (17–32)	< 0.001
Organ failures on day 3 (0 to 6) (*n* = 882)	1 (0–3)	3 (2–4)	1 (0–2)	< 0.001
CLIF‐C‐ACLF on day 3 (*n* = 698)	52 (44–60)	55 (45–64)	52 (44–60)	0.11
MELD on day 3 (*n* = 542)	27 (19–36)	30 (25–36)	23 (16–30)	< 0.001
In‐hospital outcomes				
All‐cause mortality	296 (33.0%)	5 (5.2%)	291 (36.3%)	< 0.001
ICU LOS (days)	6 (3–12)	8 (4–22)	5 (3–11)	< 0.001
Hospital LOS (days) (*n* = 896)	15 (9–27)	18 (9–35)	15 (9–26)	0.05
Year of enrollment				< 0.001
2010–2013	413 (46.0%)	60 (61.9%)	353 (44.1%)	
2014–2017	485 (54.0%)	37 (38.1%)	448 (55.9%)	

Abbreviations: AKI, acute kidney injury; BMI, body mass index; CLIF‐C‐ACLF, Chronic Liver Failure Acute‐on‐Chronic Liver Failure score; HCV, hepatitis C virus; HE, hepatic encephalopathy (West Haven criteria); ICU, intensive care unit; INR, international normalised ratio; LOS, length‐of‐stay; LT, liver transplant; MELD, Model for End‐stage Liver Disease; PF, arterial oxygen pressure/inspired oxygen fraction ratio; RRT, renal replacement therapy; sCr, serum creatinine.

On ICU day 1, patients who were transplanted had worse liver function (median INR and bilirubin of 2.4 vs. 1.9 and of 15.0 vs. 4.2 mg/dL, respectively; *p* < 0.001 for both), more severe hyperlactatemia (median lactate of 3.7 vs. 2.5 mmol/L; *p* < 0.001), and more hyponatremia (median sodium of 134 vs. 135 mmol/L; *p* = 0.006) than others (Table 2).

Transplanted patients had more AKI on ICU days 1 (68.0% vs. 56.5%; *p* = 0.031) and 3 (67.0% vs. 53.2%; *p* = 0.012) than others.

Patients who underwent LT had higher median number of organ failures or greater ACLF grading on ICU days 1 (3 vs. 2% and 15.7% for grade 3 vs. 1.7% for grade 0, respectively; *p* < 0.001 for both) and 3 (3 vs. 1% and 24.1% for grade 3 vs. 2.1% for grade 0, respectively; *p* < 0.001 for both) than others (Tables 2 and S2).

While transplanted patients had higher median MELD scores on ICU days 1 (34 vs. 25) and 3 (30 vs. 23; *p* < 0.001 for both) than others, CLIF‐C‐ACLF scores on ICU days 1 and 3 were similar among these subgroups (Table 2).

In‐hospital all‐cause mortality was lower among transplanted patients in comparison to others (5.2% vs. 36.3%; *p* < 0.001). Additionally, transplanted patients were more frequently included in the older period of 2010–2013 (vs 2014–2017) than others (61.9% vs. 44.1%; *p* < 0.001) (Table 2).

### 3.5 Univariable Analysis: Associations With In‐Hospital Transplant‐Free Survival

Patients who survived the hospital stay without LT had a higher prevalence of ALD (alone or with hepatitis C) than others (54.7% vs. 44.6%; *p* = 0.003). Moreover, on ICU day 1, spontaneous survivors had less grade 3/4 HE (31.4% vs. 41.5%; *p* = 0.002), less need for ventilation (49.4% vs. 61.1%; *p* < 0.001), less requirement for vasopressors (31.6% vs. 58.2%; *p* < 0.001), better liver function (median INR and bilirubin of 1.7 vs. 2.3 and of 3.4 vs. 8.6 mg/dL, respectively; *p* < 0.001 for both), less severe hyperlactatemia (median lactate of 2.2 vs. 3.2 mmol/L; *p* < 0.001), and less acidosis (median pH of 7.36 vs. 7.32; *p* < 0.001) than others (Table 3).

**TABLE 3:** Baseline characteristics and outcomes of critically ill patients with cirrhosis stratified by transplant‐free survival status.

Characteristic	Total (*n* = 898)	In‐hospital TFS (*n* = 510)	In‐hospital LT or death (*n* = 388)	*p*
Age (years) (*n* = 897)	57 (49–64)	57 (48–63)	57 (50–64)	0.46
Sex (male)	539 (60.0%)	303 (59.4%)	236 (60.8%)	0.67
BMI (kg/m^2^) (*n* = 590)	28 (24–33)	28 (24–34)	28 (25–32)	0.35
Cirrhosis aetiology				0.001
Alcohol	367 (40.9%)	222 (43.5%)	145 (37.4%)	
HCV	94 (10.5%)	59 (11.6%)	35 (9.0%)	
Alcohol + HCV	85 (9.5%)	57 (11.2%)	28 (7.2%)	
Others	352 (39.2%)	172 (33.7%)	180 (46.4%)	
Hepatocellular carcinoma	72 (8.0%)	36 (7.1%)	36 (9.3%)	0.23
Prior dialysis (*n* = 895)	41 (4.6%)	25 (4.9%)	16 (4.1%)	0.58
Precipitant event				0.23
Infection	357 (39.8%)	216 (42.4%)	141 (36.3%)	
Bleeding	74 (8.2%)	44 (8.6%)	30 (7.7%)	
Infection + bleeding	76 (8.5%)	42 (8.2%)	34 (8.8%)	
Other	391 (43.5%)	208 (40.8%)	183 (47.2%)	
General organ function and support on day 1				
Grade 3/4 HE	321 (35.7%)	160 (31.4%)	161 (41.5%)	0.002
Ventilation	489 (54.5%)	252 (49.4%)	237 (61.1%)	< 0.001
PF ratio (mmHg) (*n* = 564)	200 (137–280)	214 (145–306)	186 (123–266)	0.004
Vasopressors (*n* = 853)	370 (43.4%)	150 (31.6%)	220 (58.2%)	< 0.001
MAP (*n* = 597)	58 (50–66)	60 (51–68)	55 (49–64)	0.001
INR (*n* = 880)	1.9 (1.5–2.5)	1.7 (1.4–2.1)	2.3 (1.8–3.0)	< 0.001
Bilirubin (mg/dL) (*n* = 878)	4.9 (2.1–11.8)	3.4 (1.5–7.5)	8.6 (3.4–19.6)	< 0.001
Lactate (mmol/L) (*n* = 694)	2.6 (1.7–4.9)	2.2 (1.5–3.8)	3.2 (2.0–6.4)	< 0.001
Sodium (mmol/L) (*n* = 753)	135 (132–139)	135 (132–139)	135 (131–139)	0.22
pH (*n* = 673)	7.34 (7.26–7.40)	7.36 (7.28–7.41)	7.32 (7.21–7.39)	< 0.001
Kidney function and support on days 1–3				
sCr on day 1 (mg/dL) (*n* = 891)	1.69 (1.06–3.04)	1.51 (0.94–2.74)	2.01 (1.21–3.36)	< 0.001
AKI on day 1 (*n* = 891)	515 (57.8%)	262 (51.8%)	253 (65.7%)	< 0.001
sCr on day 3 (mg/dL) (*n* = 727)	1.38 (0.88–2.18)	1.28 (0.82–1.99)	1.53 (1.00–2.43)	0.002
AKI on day 3 (*n* = 727)	410 (55.0%)	204 (48.0%)	206 (64.2%)	< 0.001
RRT on days 1–3	249 (27.7%)	110 (21.6%)	139 (35.8%)	< 0.001
Severity of disease on days 1–3				
Organ failures on day 1 (0 to 6)	2 (1–3)	1 (1–2)	3 (2–4)	< 0.001
CLIF‐C‐ACLF on day 1 (*n* = 844)	56 (49–64)	54 (46–62)	59 (52–65)	< 0.001
MELD on day 1 (*n* = 870)	27 (19–35)	23 (16–30)	32 (24–39)	< 0.001
Organ failures on day 3 (0 to 6) (*n* = 882)	1 (0–3)	1 (0–2)	2 (1–3)	< 0.001
CLIF‐C‐ACLF on day 3 (*n* = 698)	52 (44–60)	50 (44–58)	55 (47–63)	< 0.001
MELD on day 3 (*n* = 542)	27 (19–36)	23 (15–31)	32 (25–40)	< 0.001
In‐hospital outcomes				
ICU LOS (days)	6 (3–12)	5 (3–10)	6 (3–14)	0.005
Hospital LOS (days) (*n* = 896)	15 (9–27)	15 (9–25)	16 (8–29)	0.44
Year of enrollment				0.31
2010–2013	413 (46.0%)	242 (47.5%)	171 (44.1%)	
2014–2017	485 (54.0%)	268 (52.5%)	217 (55.9%)	

Abbreviations: AKI, acute kidney injury; BMI, body mass index; CLIF‐C‐ACLF, Chronic Liver Failure Acute‐on‐Chronic Liver Failure score; HCV, hepatitis C virus; HE, hepatic encephalopathy (West Haven criteria); ICU, intensive care unit; INR, international normalised ratio; LOS, length‐of‐stay; MELD, Model for End‐stage Liver Disease; PF, arterial oxygen pressure/inspired oxygen fraction ratio; RRT, renal replacement therapy; sCr, serum creatinine; TFS, transplant‐free survival.

Spontaneous survivors had less AKI on ICU days 1 (51.8% vs. 65.7%; *p* < 0.001) and 3 (48.0% vs. 64.2%; *p* < 0.001) and less use of RRT on ICU days 1–3 (21.6% vs. 35.8%; *p* < 0.001) than others (Table 3).

Spontaneous survivors showed a lower overall severity of disease than others, detailed as follows: lower median number or lower ACLF grading on both ICU days 1 (1 vs. 3% and 82.2% for grade 0 vs. 35.8% for grade 3, respectively; *p* < 0.001 for both) and 3 (1 vs. 2% and 77.7% for grade 0 vs. 26.8% for grade 3, respectively; *p* < 0.001 for both); lower median CLIF‐C‐ACLF scores on ICU days 1 (54 vs. 59) and 3 (50 vs. 55; *p* < 0.001 for both); and lower median MELD scores on ICU days 1 (23 vs. 32) and 3 (23 vs. 32; *p* < 0.001 for both) (Tables 3 and S2).

### 3.6 Analysis of Competing Risks Using the Cumulative Incidence Function: Univariable Association of Renal Replacement Therapy With In‐Hospital Liver Transplant and All‐Cause Mortality

**FIGURE 1:**
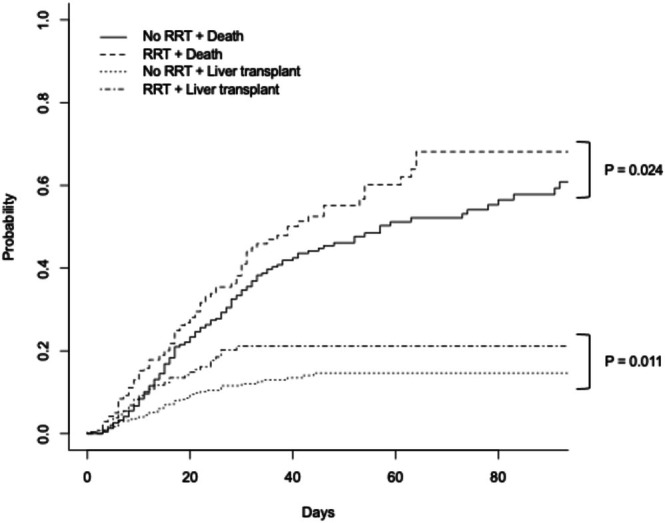
Cumulative incidence curves for index in‐hospital liver transplant or mortality with or without renal replacement therapy (univariable).

Based on the cumulative incidence curves, with in‐hospital LT and all‐cause mortality as competing events, RRT on days 1–3 was associated with both a higher likelihood of LT (*p* = 0.011) and mortality (*p* = 0.024) (Figure 1).

### 3.7 Competing Risks Using the Fine and Gray Approach: Multivariable Associations With In‐Hospital Liver Transplant and All‐Cause Mortality

Using standard adjusted Cox regression (single outcome), RRT on ICU days 1–3 was associated with higher hazard of LT (Model 1: aHR (95% CI) of 1.62 (1.07–2.44); *p* = 0.021). This association remained significant when using all‐cause mortality as a competing event (Model 4: aHR (95% CI) of 1.54 (1.02–2.32); *p* = 0.039) (Table 4).

**TABLE 4:** Multivariable analysis: Associations between renal replacement therapy and outcomes.

Characteristic	Hazard ratio (95% CI)	*p*
*Model 1: associations with in‐hospital liver transplant*		
Alcohol (vs. other aetiology)	0.52 (0.35–0.80)	0.002
Organ failures on day 1 (except kidney failure)^a^	1.35 (1.16–1.57)	< 0.001
RRT on days 1–3	1.62 (1.07–2.44)	0.021
Year 2014–2017 (vs. 2010–2013)	0.62 (0.41–0.93)	0.021
*n* included = 881 (16 excluded due to death in the first 3 days of stay, one patient excluded due to missing date or censoring); *n* events = 97; *p* < 0.001		
*Model 2: associations with in‐hospital all‐cause mortality*		
Alcohol versus other aetiology	0.89 (0.70–1.12)	0.34
Organ failures on day 1 (except kidney failure)^a^	1.33 (1.21–1.46)	< 0.001
RRT on days 1–3	1.22 (0.95–1.56)	0.13
Year 2014–2017 (vs. 2010–2013)	1.47 (1.16–1.87)	0.002
*n* included = 877 (16 excluded due to death in the first 3 days of stay, 5 patients excluded due to missing date or censoring); *n* events = 279; *p* < 0.001		
*Model 3: associations with in‐hospital transplant‐free survival*		
Alcohol versus other aetiology	1.30 (1.09–1.55)	0.004
Organ failures on day 1 (except kidney failure)^a^	0.71 (0.66–0.77)	< 0.001
RRT on days 1–3	0.93 (0.75–1.15)	0.48
Year 2014–2017 (vs. 2010–2013)	1.12 (0.94–1.33)	0.21
*n* included = 879 (16 excluded due to death in the first 3 days of stay, 3 patients excluded due to missing date or censoring); *n* events = 510; *p* < 0.001		
*Model 4: associations with in‐hospital liver transplant with all‐cause mortality as a competing event*		
Alcohol versus other aetiology	0.53 (0.35–0.81)	0.003
Organ failures on day 1 (except kidney failure)^a^	1.28 (1.12–1.47)	0.001
RRT on days 1–3	1.54 (1.02–2.32)	0.039
Year 2014–2017 (vs. 2010–2013)	0.55 (0.36–0.82)	0.004
*n* included = 881 (16 excluded due to death in the first 3 days of stay, 1 patient excluded due to missing date or censoring)		
*Model 5: associations with in‐hospital all‐cause mortality with liver transplant as a competing event*		
Alcohol versus other aetiology	0.95 (0.75–1.19)	0.65
Organ failures on day 1 (except kidney failure)^a^	1.32 (1.21–1.44)	< 0.001
RRT on days 1–3	1.15 (0.90–1.47)	0.25
Year 2014–2017 (vs. 2010–2013)	1.70 (1.35–2.15)	< 0.001
*n* included = 881 (16 excluded due to death in the first 3 days of stay, 1 patient excluded due to missing date or censoring)		

Abbreviations: CI, confidence interval; RRT, renal replacement therapy.

^a^ Number of organ failures observed on day 1 excluding kidney failure (minimum of 0 and maximum of 5). Organ failures were defined by Chronic Liver Failure Sequential Organ Failure Assessment score.

Conversely, RRT on ICU days 1–3 was not associated neither with all‐cause mortality (Model 2: aHR (95% CI) of 1.22 (0.95–1.56); *p* = 0.13) nor with TFS (Model 3: aHR (95% CI) of 0.93 (0.75–1.15); *p* = 0.48). This lack of association between RRT on ICU days 1–3 and all‐cause mortality was also observed when using LT as a competing event (Model 5: aHR (95% CI) of 1.15 (0.90–1.47); *p* = 0.25) (Table 4).

When considering only patients on continuous RRT (197 out of 249 patients on any RRT), the associations between RRT on ICU days 1–3 and LT (aHR (95% CI) of 1.64 (1.06–2.53); *p* = 0.026) or all‐cause mortality (aHR (95% CI) of 1.25 (0.97–1.61); *p* = 0.09) were similar.

ALD was associated with lower hazard of LT both in standard regression and after using mortality as a competing event (Model 4: aHR (95% CI) of 0.53 (0.35–0.81); *p* = 0.003). Moreover, higher median number of organ failures on ICU day 1 (except for kidney failure) was also associated with higher hazard of LT (Model 4: aHR (95% CI) of 1.28 (1.12–1.47); *p* = 0.001) or all‐cause mortality (Model 5: aHR (95% CI) of 1.32 (1.21–1.44); *p* < 0.001) both in standard regression and following competing risk regression. Finally, inclusion during the years 2014–2017 (vs. 2010–2013) was associated with lower hazard of LT both in standard regression and when accounting for all‐cause mortality as a competing event (Model 4: aHR (95% CI) of 0.55 (0.36–0.82); *p* = 0.004). Conversely, such inclusion period was associated with higher hazard of all‐cause mortality both in standard regression and after using LT as competing event (Model 5: aHR (95% CI) of 1.70 (1.35–2.15); *p* < 0.001) (Table 4).

## 4 Discussion

### 4.1 Main Findings and Comparisons With Previous Literature

Using a large cohort of patients with cirrhosis admitted to the ICUs of 2 LT centers in North America from 2010 to 2017, we showed that RRT was offered to 27.7% of patients in any of the first 3 days of ICU stay. Patients who required RRT were sicker as they had higher median CLIF‐C‐ACLF and MELD scores than others. Based on both standard and competing risk multivariable analysis, following adjustment for significant confounders (aetiology, severity of disease, and year of enrollment), being on RRT on days 1–3 was independently associated with a higher hazard of getting a LT. Conversely, being on RRT on days 1–3 was not associated with all‐cause mortality.

To the best of our knowledge, few studies have examined the association between RRT and survival specifically among critically ill patients with cirrhosis. In a study by Baudry et al., among 149 patients with cirrhosis and septic shock, having received RRT was associated with higher ICU and 1‐year all‐cause mortality [8]. In another study by O'Brien et al., among 444 patients with AKI and liver dysfunction (defined as serum bilirubin ≥ 2 mg/dL), RRT intensity was not associated with 90‐day all‐cause mortality [25]. In a different study by Liao et al., among 2138 patients with cirrhosis and AKI, higher severity of AKI (including the need for RRT) was associated with higher 30‐day all‐cause mortality [10].

In studies that assessed the association between RRT initiation and survival among general critically ill patients, either the proportion of patients with cirrhosis included was very low to draw any meaningful conclusions, or these patients were excluded from these large trials [20, 26].

Overall, it remains difficult to compare the results of our study with those reported in the previous literature as the baseline characteristics of patients and the selected endpoints have been substantially different. Moreover, RRT has been mainly studied as a surrogate for the severity of AKI or overall acuity (e.g., MELD or CLIF‐C‐ACLF scores), thus precluding the study of its potential independent association with core clinical outcomes. Interestingly, in our cohort, although patients on early RRT had higher severity of disease (CLIF‐C‐ACLF and MELD scores), RRT per se was not independently associated with mortality. This may suggest that RRT may have a potential impact on clinical outcomes outside the realm of being only a marker of the severity of AKI.

Additionally, there has been a lack of studies weighing the competing behaviour between LT and all‐cause mortality, especially among critically ill patients with cirrhosis [12, 27]. This sort of analysis may be especially important for clinicians as they often struggle with decisions around the level and duration of organ support they should offer to these patients, especially while no LT eligibility or availability decision has been reached. In this sense, our results suggest that patients with cirrhosis admitted to the ICU on RRT as early as in the first 3 days of ICU stay may have a higher likelihood of being transplanted. Furthermore, as observed in our cohort, this effect may be relevant even in a context of a low LT rate (10.8%) and high median time to LT (12 days). Potentially, RRT may help to control severe acid–base or electrolyte derangements; additionally, it may be deployed as an adjunctive treatment of hyperammonemia or fluid overload [28, 29].

In our cohort, ALD was independently associated with lower hazard of getting a LT, but it was not associated with all‐cause mortality. While we could not retrieve data on patient‐level specific criteria for LT selection, this could reflect center‐specific attitudes towards alcohol abstinence before LT. In fact, some length of alcohol abstinence, even if less than the classical 6‐month rule, was a requirement in both centers at the time. Moreover, we could not ascertain how many of these patients had alcoholic hepatitis, a condition with high short‐term mortality that has been more recently and increasingly recognised as an indication for emergent LT [12, 27, 30].

In our study, a higher number of extra‐renal organ failures was independently associated with higher hazard of either LT or all‐cause mortality. This finding is in line with previous literature reporting that ACLF portends high mortality, while some of these patients may benefit from LT [21, 22]. Interestingly, while higher MELD score on days 1 or 3 was associated with being transplanted, CLIF‐C‐ACLF on days 1 or 3 was not. This finding may reflect how clinicians at both centers were selecting critically ill patients with cirrhosis for LT at the time, within national allocation systems based largely on MELD score. However, recent studies have shown that MELD score may underestimate the risk of mortality among patients with ACLF; in fact, CLIF‐C‐ACLF score may better quantify their short‐term prognosis [31].

In our cohort, being included during the latest period (2014–2017) was independently associated with a lower hazard of being transplanted and a higher hazard of dying in comparison to being included during the oldest period (2010–2013). In fact, the LT rate was lower in 2014–2017 than in 2010–2013 (7.6% vs. 14.5%). Thus, this difference in the proportion of patients selected for LT at each center could have influenced the change in the risk profile of LT or death between the time periods. However, we did not have further data to clarify those transplant eligibility decisions at each institution across time, which often tend to be complex and possibly subject to some degree of heterogeneity.

### 4.2 Limitations, Strengths, and Future Directions

The results of our study need to be interpreted in the context of the following limitations. First, this was a retrospective analysis of a large sample of critically ill patients with cirrhosis; therefore, it is prone to selection bias and confounding. We did not account for patients denied critical care or only eligible for palliative care. The large size of our multicenter sample may have helped to mitigate such bias.

Two, the definition of AKI used may have underestimated this diagnosis. However, AKI was present in 57.8% of patients on day 1, which is a substantial proportion echoing previous studies. More importantly, in this study we were concentrated on the use of RRT, the higher severity of the AKI spectrum. In fact, 91.6% of patients with RRT on days 1–3 had AKI, which kind of validates our definition.

Three, the specifics of RRT prescription may have varied across centers and time periods as clinical practice has been evolving over time. In fact, the acute dialysis quality initiative has been improving the benchmark targets for RRT prescription in different clinical scenarios [32, 33]. Nevertheless, both hospitals involved in this study have been striving to be at the forefront of the implementation of updated guidelines on the management of AKI in patients with cirrhosis.

Four, the potential impact of RRT on these patients' outcomes would be ideally studied by performing a randomised clinical trial, but such a study may be difficult to design and implement due to ethical, logistical, and cost issues. Moreover, in large trials designed to study RRT efficacy and safety in general critically ill patients, those with cirrhosis have often been under‐recruited or excluded from such studies altogether. Thus, our multicenter retrospective cohort study, with competing risk analysis for LT and all‐cause mortality, albeit with inherent shortcomings, may have been a fair initial approach to this topic with the potential to inform and promote a future trial.

Five, confounding could have arisen from the lack of data on specific LT eligibility criteria being considered over time at both centers. In fact, transplant criteria for critically ill patients with cirrhosis have been evolving over time as more studies have documented post‐transplant reasonably good results even among those with at least 3 organ failures [21, 22].

Taken all these limitations into consideration, we believe our study adds to the literature by providing a real‐world characterisation of RRT deployment among critically ill patients with cirrhosis. Furthermore, it shows how the association of RRT with clinically important outcomes may be studied, namely by weighing the competing risk between short‐term LT and all‐cause mortality. Futures studies could build on our analysis by including more recent patients from different global regions, ideally in a close to or actual randomised controlled trial setting.

## 5 Conclusions

Among critically ill patients with cirrhosis, early RRT, while offered more often to the sickest ones, may be associated with a higher likelihood of undergoing LT during the index hospital stay, but not with all‐cause mortality. Further prospective studies should clarify how RRT may influence these patients' outcomes.

## Author Contributions

Filipe S. Cardoso: study concept and design; acquisition of data; analysis and interpretation of data; statistical analysis; drafting of the manuscript; critical revision of the manuscript for important intellectual content. Minjee Kim: acquisition of data; critical revision of the manuscript for important intellectual content. Beverley Kok: acquisition of data; critical revision of the manuscript for important intellectual content. Richard Wunderink: acquisition of data; critical revision of the manuscript for important intellectual content. Juan G. Abraldes: analysis and interpretation of data; statistical analysis; critical revision of the manuscript for important intellectual content. Constantine J. Karvellas: analysis and interpretation of data; drafting of the manuscript; critical revision of the manuscript for important intellectual content; study supervision.

## Funding

The authors have nothing to report.

## Ethics Statement

The institutional review boards of both centers approved this study (Pro00035429, 04/02/2013, University of Alberta Health Research Ethics Board; STU00204868, 08/11/2017, Northwestern University Institutional Review Board).

## Consent

As this was a noninterventional and anonymised study, the institutional review boards of both centers waived the need for individual informed consent.

## Conflicts of Interest

The authors declare no conflicts of interest.

## Supporting information


**Table S1:** Acute‐on‐chronic liver failure grading and organ failures on intensive care unit days 1 and 3.
**Table S2:** Acute‐on‐chronic liver failure grading and in‐hospital liver transplant and transplant‐free survival.

## Data Availability

The data that support the findings of this study are available on request from the corresponding author. The data are not publicly available due to privacy or ethical restrictions.
