# Supplementary material for: Bridging Critically Ill Patients With Cirrhosis to Transplant With Renal Replacement Therapy: A Multicenter Cohort Study
Source: Liver Int. 2026 Mar 18;46(4):e70593. doi: 10.1111/liv.70593 (PMC12997015; doi:10.1111/liv.70593)
Supplement: Supplementary file 1 — Table S1: Acute‐on‐chronic liver failure grading and organ failures on intensive care unit days 1 and 3. Table S2: Acute‐on‐chronic liver failure grading and in‐hospital liver transplant and transplant‐free survival. [file LIV-46-0-s001.docx]

**Bridging critically ill patients with cirrhosis to transplant with renal replacement therapy: a multicenter cohort study**

Filipe S. Cardoso MD MSc^1^, Minjee Kim MD^2^, Beverley Kok MD^3^, Richard Wunderink MD^4^, Juan G. Abraldes MD PhD^5^, Constantine J. Karvellas MD MSc^5,6^

^1^ Transplant Unit, Curry Cabral Hospital, Nova Medical School, Lisbon, Portugal

^2^ Division of Neurocritical Care, Department of Neurology, Northwestern University Feinberg School of Medicine, Chicago, Illinois, USA

^3^ Liver Unit, Royal Free Hospital, London, UK

^4^ Department of Medicine, Northwestern University Feinberg School of Medicine, Chicago, Illinois, USA

^5^ Liver Unit, University of Alberta Hospital, Edmonton, Canada

^6^ Department of Critical Care, University of Alberta Hospital, Edmonton, Canada

**Table S1. Acute-on-chronic liver failure grading and organ failures on intensive care unit days 1 and 3.**

| ACLF grading* | Organ failures* | n (%) |
| --- | --- | --- |
| Day 1 (n=898) | | |
| 0 | 0 | 118 (13.1%) |
| 1 | 1 | 258 (28.7%) |
| 2 | 2 | 198 (22.0%) |
| 3 | ≥3 | 324 (36.0%) |
| Day 3 (n=882) | | |
| 0 | 0 | 292 (33.1%) |
| 1 | 1 | 217 (24.6%) |
| 2 | 2 | 149 (16.9%) |
| 3 | ≥3 | 224 (25.4%) |
| * European Foundation for the Study of Chronic Liver Failure. | | |

**Table S2. Acute-on-chronic liver failure grading and in-hospital liver transplant and transplant-free survival**

| ACLF grading* | Liver transplant^a^ | TFS^b^ |
| --- | --- | --- |
| Day 1 (n=898) | | |
| 0 | 2 (1.7%) | 97 (82.2%) |
| 1 | 14 (5.4%) | 191 (74.0%) |
| 2 | 30 (15.2%) | 106 (53.5%) |
| 3 | 51 (15.7%) | 116 (35.8%) |
| Day 3 (n=882) | | |
| 0 | 6 (2.1%) | 226 (77.7%) |
| 1 | 15 (6.9%) | 156 (71.9%) |
| 2 | 22 (14.8%) | 68 (45.6%) |
| 3 | 54 (24.1%) | 60 (26.8%) |
| * European Foundation for the Study of Chronic Liver Failure.  ^a^ ACLF grading on day 1 vs liver transplant: P<0.001. ACLF grading on day 1 vs TFS: P<0.001.  ^b^ ACLF grading on day 3 vs liver transplant: P<0.001. ACLF grading on day 3 vs TFS: P<0.001.  TFS: transplant-free survival. | | |
